# Critical role of extracellularly secreted neuronal pentraxin 1 in ischemic neuronal death

**DOI:** 10.1186/s12868-014-0133-3

**Published:** 2014-12-20

**Authors:** Shabarish Thatipamula, Mir Ahamed Hossain

**Affiliations:** Hugo W. Moser Research Institute at Kennedy Krieger, Baltimore, MD 21205 USA; Department of Neurology, Johns Hopkins University School of Medicine, Baltimore, MD 21205 USA; Department of Neurology, The Kennedy Krieger Institute, 707 North Broadway, Room 400-N, MD 21205 Baltimore, USA

**Keywords:** Oxygen glucose deprivation, Neuronal pentraxin 1, Conditioned medium, Neuronal death, Primary cortical neurons, Synapse, AMPA receptor GluR1

## Abstract

**Background:**

Developing brain is highly susceptible to hypoxic-ischemic injury leading to severe neurological disabilities in surviving infants and children. Previously we reported induction of neuronal pentraxin 1 (NP1) in hypoxic-ischemic injury in neonatal brain and NP1 co-localization with the excitatory AMPA receptors GluR1 at the synaptic sites. However, how NP1 contributes to hypoxic-ischemic neuronal injury is not completely understood.

**Results:**

Here we report that extracellular secretion of NP1 is required for ischemic neuronal death. Primary cortical neurons at days in vitro (DIV) 12 were subjected to oxygen glucose deprivation (OGD), an *in vitro* model of ischemic stroke, for different time periods (2–8 h). Oxygen glucose deprivation showed characteristic morphological changes of dying cells, OGD time-dependent induction of NP1 (2-4-fold) and increased neuronal death. In contrast, the NP1-KO cortical neurons were healthy and showed no sign of dying cells under similar conditions. NP1gene silencing by NP1-specific small interfering RNA (NP1-siRNA) protected cortical neurons from OGD-induced death. Conditioned media (CM) collected from OGD exposed WT cortical cultures caused neurotoxicity when added to a subset of DIV 12 normoxia control WT cortical cultures. In contrast, CM from OGD-exposed NP1-KO cultures did not induce cell toxicity in control WT cultures, suggesting a role for extracellular NP1 in neuronal death. However, NP1-KO neurons, which showed normal neuronal morphology and protection against OGD, sustained enhanced death following incubation with CM from WT OGD-exposed cultures. Western blot analysis of OGD exposed WT CM showed temporal increase of NP1 protein levels in the CM. Most strikingly, in contrast to NP1-KO CM, incubation of normal cortical cultures with CM from OGD exposed NP2-KO cultures showed neurotoxicity similar to that observed with CM from OGD exposed WT neuronal cultures. Western immunoblotting further confirmed the increased presence of NP1 protein in OGD-exposed NP2-KO CM. Live immunofluorescence analysis show intense cell surface clustering of NP1 with AMPA GluR1 receptors.

**Conclusions:**

Collectively, our results demonstrate that extracellular release of NP1 promote hypoxic-ischemic neuronal death possibly *via* surface clustering with GluR1 at synaptic sites and that NP1, not its family member NP2, is involved in the neuronal death mechanisms.

## Background

Neuronal injury occurring with cerebral hypoxia-ischemia (HI) has been attributed to overstimulation of N-methyl-D-aspartate (NMDA) and α-amino-3-hydroxy-5-methyl-4-isoxazole-propionic acid (AMPA) subtypes of glutamate receptors [1–4], oxidative stress, and activation of intrinsic program of apoptotic cell death [5]. One of the proteins that is induced under hypoxic-ischemic stress and initiates neuronal death program is ‘neuronal pentraxin 1′ (NP1) [6–8]. However, how NP1 contributes to neuronal death is not completely understood. NP1 is predominantly expressed in the central nervous system [9–12] and belongs to the ‘long pentraxins’ family of proteins; NP1, neuronal activity-regulated pentraxin (Narp; also called NP2), and neuronal pentraxin receptor (NPR) [9,12,13]. Neuronal pentraxins have high homology among human, mouse and rats [14]. The pentraxins have several structural and functional characteristics to form side-to-side and head-to-head multimeric aggregates [13,15,16] and the ability to bind other proteins *via* a lectin-like domain.

Proposed functions of NPs include modulating synaptic uptake, synapse formation, and synaptic remodeling [9,17]. NP2 has been reported to mediate synaptic clustering of AMPA glutamate receptors [18,19]. In our previous studies, we have shown induction of NP1 in neonatal mice brain following HI and injury to the cerebral cortex and hippocampal CA3 and CA1 brain regions [7,20,21]. We found that the increase in NP1 induction occurs before the actual cell death, consistent with a role for NP1 in the injury mechanisms. We also found that NP1 co-localizes with AMAP GluR1 receptors and enhanced GluR1 membrane insertion at the synaptic sites as evident by NP1-GluR1-PSD-95 co-clustering following OGD exposure [22]. It is known that various cell death mechanisms require *de novo* synthesis of both RNA and lethal proteins [5,23], and low neuronal activity triggers the intrinsic program of apoptotic cell death in mature neurons [5]. However, how induction of NP1 expression leads to the propagation of neuronal death or survival of neurons in the absence of NP1 expression is not completely understood. Here, we report that the extracellular secretion of NP1 is required to induce neuronal death in primary cortical neurons subjected to oxygen glucose deprivation (OGD) possibly through co-clustering with APMA GluR1 receptors at synaptic sites and enhanced excitotoxicity. Our findings suggest that blockade of NP1 induction and its extracellular release may be therapeutically relevant against hypoxic-ischemic injury in neonatal brain.

## Methods

### Embryonic cortical neuronal culture

The Johns Hopkins University Institutional Animal Care and Use Committee approved all animal protocols used; they complied with the US NIH Guide for the Care and Use of Laboratory Animals. Primary cortical neuronal cultures were prepared from embryonic day 16 (E16) wild-type (WT) and NP1-knockout (NP1-KO) mice as described previously [7]. NP1 knockout mice were provided by Dr. Paul Worley, Dept. of Neuroscience, School of medicine, Johns Hopkins University, Baltimore, MD, USA. Primary cortical neurons were grown in a culture medium consisting of Neurobasal™ medium (Invitrogen, Carlsbad, CA, USA), 2% B27 supplement (Invitrogen), 2-mM L-glutamine, and 1% penicillin-streptomycin as described previously [7]. At 3 days in vitro (DIV), one-third of the media was replaced with fresh medium (without L-glutamine) containing cytosine arabinofuranoside (AraC, 5 μM; Sigma, St. Louis, MO, USA) to arrest the growth of non-neuronal cells. Experiments were conducted at DIV 12, when cultures consisted primarily of neurons (>95% MAP-2 immunoreactive cells) (MAP-2; Chemicon, Temecula, CA, USA).

### Induction of OGD, modeled *in vitro*, using cultured primary cortical neurons

To induce oxygen glucose deprived conditions, cultured cortical neurons at DIV 12 were exposed to OGD as described previously [6,22,24]. Briefly, neurons were placed in glucose-free Earl’s balanced salt solution (EBSS) and then exposed to humidified 95% N_2_/5% CO_2_ using anaerobic modular incubator chambers (Billups-Rothenberg, Del Mar, CA, USA) for different time periods (2–8 h). Control cultures were incubated with EBSS with glucose and incubated in humidified 95% air/5% CO_2_ for the same duration. After indicated periods of OGD, cells were washed with ice-cold PBS and harvested to examine various biochemical and morphological end points.

### SDS-PAGE and western blot analyses

SDS-PAGE and immunoblotting were performed according to the method as described previously [7,25,26]. Total proteins (20–30 μg) were diluted in Laemmli buffer containing 2-mercaptoethanol, heated to 95°C for 5 min, separated on a 4-20% gradient Tris-glycine precast gel (Invitrogen) at 120 V for 1.5 h. Blots were incubated with primary antibodies specific for NP1 (1:500, BD Transduction Laboratories, Tamecula, CA, USA). HRP (horseradish peroxidase)-conjugated secondary antibodies (GE Healthcare, Piscataway, NJ, USA) were used at 1:10000 dilutions for 1 h at room temperature. The HRP reaction product was visualized using an ECL Western blotting detection kit (GE healthcare). Image films were scanned in gray scale (HP Scanjet G4010) at a high resolution as TIFF files. Immunoreactive protein bands corresponding to the correct molecular mass of target protein were quantified by drawing rectangle around the individual band and the intensity was measured by densitometry using NIH ImageJ software. Values were normalized to internal standard actin, which also serve as a loading control, to make relative comparisons.

### Assessment of cell viability/toxicity

Immediately after the indicated periods of OGD exposure, cell viability and cell death was determined by independent and complementary methods as described previously [6,7,22,24].

#### MTT assay

Mitochondrial dehydrogenase activity cleaves 3-(4,5-dimethylthiazol-2-yl)-2,5-diphenyl tetrazolium bromide (MTT; Sigma), which is considered as a biochemical index for cellular viability. A quantitative colorimetric assay of MTT [27] used to determine cell survival as described previously [7,25]. The results were expressed as a percentage of control cultures viability.

#### LDH assay

Lactate dehydrogenase (LDH) activity released in the media after OGD exposure was measured using the CytoTox96 Non-radioactive Cytotoxicity Assay kit (Promega, Madison, WI, USA) as described previously [7,28]. Percent cell death was determined using the formula: % cytotoxicity = OGD-induced LDH release (OD_490_)/maximum LDH release (OD_490_) after correcting for baseline absorbance of LDH release at 490 nm.

#### TUNEL staining

The DeadEnd Fluorometric TUNEL System (Promega) was used to detect cell death in cultured primary cortical neurons exposed to OGD (2-8 h) as described previously [29,30]. This method allows direct detection of nuclear DNA fragmentation, an important biochemical hallmark of cell death, by catalytically incorporating fluorescein-12-dUTP at 3′-OH DNA ends. Primary cortical cultures grown on cover slips were processed according to manufacturers’ instructions. Negative controls were performed under identical conditions except for the omission of terminal deoxynucleotidyl transferase (TdT) from the reaction buffer. Fluorescein fluorescence was visualized in a fluorescence microscope (Carl Zeiss Axioplan 1) with an excitation at 485 nm and an emission at 535 nm. DAPI fluorescence (blue) was visualized with an excitation and emission filters at 365 nm and 450 nm, respectively.

### Short interference RNA (siRNA) directed against NP1 mRNA

For NP1 gene silencing experiments, we have used *Ntpx1* specific siRNA constructs (5′-AATTCTTCCAGCCAAACCAAC-3′) (construct #3) (5-AAGAACGACACAGAGGAAAGG-3′) (construct #5) generated using Silencer™ siRNA construction kit (Cat #1620) (Ambion, Inc. Austin, TX, USA) and the commercially available control scramble siRNA (SsiRNA) following methods described previously [8]. The oligodeoxyribonucleotide sequences exhibited no similarity to any other known mammalian genes as determined by BLAST. Experimental treatments were initiated ~ 48 h after transfection. Using siRNA specific for NP1, we have achieved >90% reduction in NP1 protein levels compared to control SsiRNA.

### Quantification of NP1 expression by real-time PCR

Total RNA was extracted from control and OGD-exposed primary cortical cultures using TRIzol reagent (Invitrogen) according to manufacturer’s protocol. The cDNA was synthesized from 1 μg of purified total RNA using iScript™ cDNA Synthesis Kit (Bio-Rad laboratories, Richmond, CA, USA), following the manufacturer’s instructions. Quantitative real-time PCR was performed in triplicate by using iQ SYBR Green Supermix on CFX96™ Real-Time System (Bio-Rad) as described previously [6]. The mRNA level was normalized by housekeeping gene HPRT [31]. The primers set used for NP1 (Acc. No. NM_008730.2) were 5′-GCT GCG AGA GCC AGA GCA CC-3′ (sense) and 5′-TTG CCC GAG TTG GCT GAG CG-3′ (anti-sense), and for HPRT were 5′-CCT GGC GTC GTG ATT AGT GAT G-3′ (sense) and 5′-CAG AGG GCT ACA ATG TGA TGG C-3′ (anti-sense).

### Immunofluorescence

Live double-immunofluorescence staining of primary cortical cultures (DIV 12) with NP1 was done as described previously [7,22]. Briefly, cortical neurons, grown on coverslips, following exposure to OGD (4 h) were live labeled with both anti-NP1 (1:100; BD Transduction Laboratories) and anti-GluR1 (1:100; Millipore) by adding directly to the medium and further incubated for 45 min at 37°C. Neurons were then fixed with 3.7% formaldehyde, and permeabilized cells were stained with anti-mouse Alexa Fluor 568 (red, NP1) and anti-rabbit Alexa fluor 488 (green, GluR1)-conjugated secondary antibodies (Invitrogen). Slides were coverslipped with prolong mounting medium containing DAPI (blue) (Molecular Probes, Eugene, OR, USA) to stain nuclei. Immunofluorescence was visualized using an inverted fluorescence microscope (Olympus IX51fitted with DP2-DSW-V3.2 application software) at 10 × and ZEISS Axioimager M2 (AxioVision SE64 Rel.4.8.1 application software) at 100 × magnification.

### Statistical analysis

Statistics were performed using GraphPad Prism software, Version 5.01. For one experimental and one control group, two-tailed Student’s *t*-test was used to determine if differences exist between means. Comparisons involving multiple groups were done by ANOVA, followed by Bonferroni/Dunn post-hoc test where appropriate. Significance level was assigned at P < 0.05.

## Results

### Induction of NP1 in primary cortical neurons exposed to oxygen glucose deprivation

**Figure 1:**
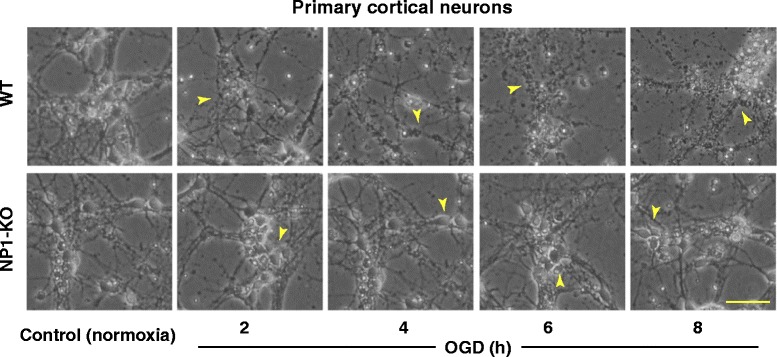
**The OGD time-dependent progression of cytotoxicity in primary cortical neurons.** Primary cortical cultures at DIV 12 were submitted to OGD conditions for indicated time periods as described in the Methods. Light microscopic images show morphological evidence of degenerated neurons and disintegration of processes (yellow arrow) of WT cortical neurons (upper panels) and healthy cell bodies with intact processes of NP1-KO neurons (lower panels) observed following OGD exposure as compared to respective normoxia control neurons. Scale bar, 100 μm.

Primary cortical neuronal cultures at DIV12 were exposed to OGD for indicated times (2–8 h). Control cultures were incubated with EBSS containing glucose and exposed to humidified 95% air/5% CO_2_ for the same duration of time. Light microscopic analysis showed that control neurons were healthy and retained normal morphology, as indicated by larger size, phase brightness and intact processes. Whereas, OGD exposed neurons showed characteristic morphological changes of dying cells, which were round, smaller and translucent with disintegration of processes and cell bodies compared to control normoxic neurons (Figure 1). In contrast, NP1-KO cortical cultures retained normal neuronal morphology; intact processes and healthy cell bodies against OGD (2–8 h), similar to normoxia control cultures (Figure 1). This *in vitro* OGD model was used in subsequent experiments to determine the role of NP1 and its specific requirement for ischemic neuronal death.

**Figure 2:**
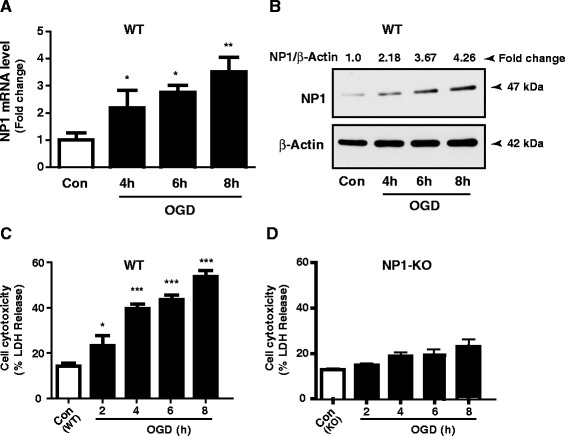
**The NP1 induction is associated with OGD-induced cortical neuronal death. A)** Total cellular RNA was extracted and NP1 mRNA expression levels were analyzed by RT-qPCR. Data show relative quantification of *Nptx1* expression at different time periods of OGD exposure. Fold induction is the ratio of NP1 to internal control HPRT, which remained stable throughout the OGD period (mean ± SEM, n = 6; *p < 0.05, **p < 0.01). **B)** Total cellular protein was analyzed by SDS-PAGE and immunoblotted for NP1 protein using NP1-specific antibody that detected NP1-immunoreactive single band of molecular mass 47 kDa. The β-actin serves as loading control. Quantitative densitometry values normalized to β-actin (NP1/β-actin ratio, n = 6) are also shown. Representative bands are shown. **C** & **D)** OGD exposure of WT cortical neurons caused cell death, while NP1-KO neurons were protected against OGD. Quantification of cell death as indicated by LDH release showed OGD time dependent increase of LDH release at 2, 4, 6, and 8 h of OGD exposures of WT cortical neurons. LDH release remained at the control level or non-significant increase in OGD-exposed NP1-KO neurons. Data are expressed as % LDH release normalized to normoxia control (mean ± SEM, n = 8; *p < 0.05, ***p < 0.001). We found ~ 50% cytotoxicity occurred at 6 h of OGD.

Next, we asked if NP1 is induced in cultured primary cortical neurons in response to OGD. The RT-qPCR data showed OGD time-dependent increased expressions of NP1 mRNA (~4-fold; p < 0.01) compared to that in normoxia controls (Figure 2A). The increased expression of NP1 mRNA was further validated by Western blot analyses (Figure 2B). Western blot analysis of total cellular extracts revealed a NP1-specific immunoreactive protein band with apparent molecular mass of ~47 kDa consistent with the expected size of NP1 [9,17]. Quantitative densitometry values of NP1 protein normalized to β-actin (NP1/β-actin ratio, n = 6) further confirmed OGD time-dependent increase of NP1 protein levels, which reached the maximum (4-fold) at 8 h of OGD, examined. The NP1 protein levels in normoxia controls were low but detectable range.

### OGD exposure caused cytotoxicity in WT neurons, whereas, NP1-KO cortical neurons are protected against OGD

Primary cortical neuronal cultures at DIV 12 were exposed to OGD for different time periods (4, 6, and 8 h). LDH release cytotoxicity assay revealed OGD time-dependent increase in cell death in WT cortical cultures. OGD exposure (4–6 h) resulted 30-50% (p < 0.001) cell death in WT cultures (Figure 2C). In contrast, we observed very negligible change (not significant) in the release of LDH in NP1-KO neurons as compared to the WT cells following OGD exposure. Interestingly, NP1-KO cortical neurons maintained morphological integrity for, at least, 8 h of OGD exposure, which were consistent with cell cytotoxicity as determined by % LDH release (Figure 2D).

### Specific involvement of NP1 in OGD-induced neuronal death

**Figure 3:**
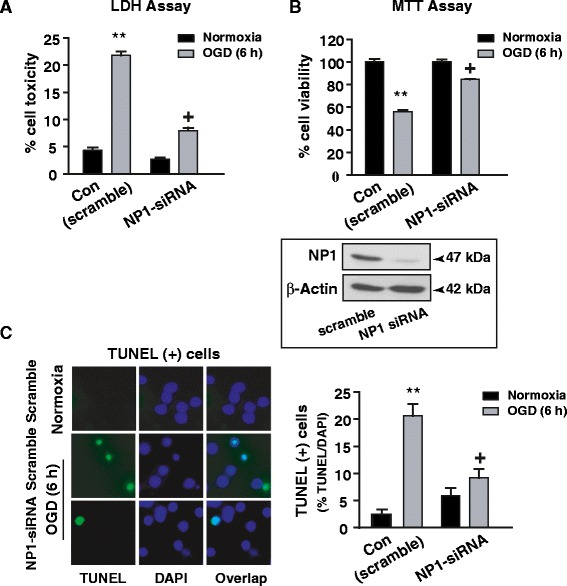
**Knockdown of NP1 by siRNA targeted against NP1 mRNA significantly protected against OGD-induced neuronal death.** We have achieved >90% reduction in NP1 protein compared to scramble siRNA (*shown in inset*). LDH cytotoxicity and MTT cell viability **(A-B)** showed significantly less cell death in NP1-siRNA transfected WT cells *vs.* non-transfected cells following exposure to OGD (6 h). Data are expressed as percent LDH release normalized to normoxia control (mean ± SEM, n = 8; **p < 0.01 compared to control normoxia; +p < 0.01compared to OGD scramble siRNA group) **C)** TUNEL staining of cortical neurons transfected with either control scramble or NP1-siRNA revealed significant increase in the number of TUNEL (+ve) cells in scramble SsiRNA transfected cultures, which was significantly reduced in NP1-siRNA transfected neurons. Data are expressed as percent TUNEL/DAPI cells (mean ± SEM, n = 8; **p < 0.01 compared to control normoxia; +p < 0.01compared to OGD scramble siRNA group).

To determine the specificity of NP1 induction in neuronal death, we transfected WT primary cortical neurons with either control scramble (SsiRNA) or NP1-siRNA to knockdown NP1 in WT neurons (Figure 3). Using our established protocol, we found that NP1-siRNA almost completely knockdown NP1 protein levels (>90%) compared to that in cells transfected with control SsiRNA (shown in inset). Here, we asked that if the NP1 induction is directly associated with neuronal death then knocking down of NP1 protein will protect cortical neurons against OGD-induced death. LDH cytotoxicity (Figure 3A), MTT cell viability (Figure 3B) assays and TUNEL (+) staining for degenerated neurons (Figure 3C) revealed that OGD exposure (6 h) resulted significant cell death (p < 0.01) in WT neurons transfected with control scramble siRNA. In contrast, primary cortical neurons transfected with NP1-siRNA, showed significantly decreased neurotoxicity and degenerated neurons (i.e. neuroprotection) when submitted to OGD exposure (6 h). Our results clearly demonstrate that NP1 is specifically involved in hypoxic-ischemic neuronal death.

### Enhanced neuronal death induced by conditioned media (CM) from OGD-exposed WT primary cortical cultures

**Figure 4:**
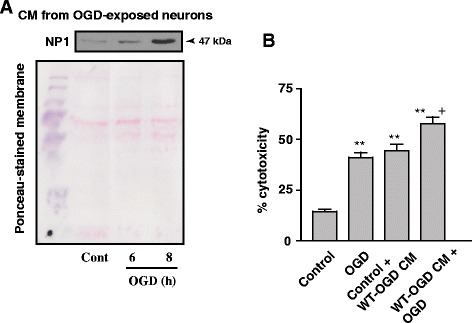
**OGD-conditioned culture media caused increased neuronal death in cortical neuronal cultures.** Primary cortical cultures at DIV 12 were exposed to OGD and conditioned media (CM) were collected, centrifuged and concentrated as described in the methods. **A)** Western blot analysis of CM from OGD-exposed WT primary cortical cultures showed OGD time-dependent increase of NP1 protein levels. **B)** This CM of OGD exposed WT cultures (WT-OGD CM) was added to a subset of control cortical cultures for 24 h (Control + WT-OGD CM). In addition, another subgroup of cells was also exposed to 6 h of OGD (WT-OGD CM + OGD). Data shown are mean ± SEM (n = 8 in each group) and repeated two times, **p < 0.01 *vs.* normoxia controls; +p < 0.01 *vs.* OGD only and Control + WT-OGD CM group.

Because of the secretory nature of NP1 [9,11–13], we asked if NP1 induction is associated with hypoxic-ischemic neuronal death, then extracellularly secreted NP1 might be also contributing to the neuronal death observed. We collected the conditioned media (CM) from control and OGD exposed WT cortical cultures (WT-OGD CM) and concentrated using Microsep™ 30K Omega Centrifugal Devices (Cat no. OD030C46; Pall Life Sciences, Ann Arbor, MI, USA). Western blot analysis of WT control and OGD-exposed CM confirmed OGD time-dependent increase of NP1 protein levels present in the WT-OGD CM (Figure 4A). After confirming the presence of NP1 in the WT-OGD CM, we added this CM to a subset of control cortical cultures at DIV 12 and incubated for additional 24 h. An additional group of the same CM-treated cultures were also exposed to OGD (6 h). LDH release cytotoxicity assay revealed that addition of WT-OGD CM to control normoxia cultures resulted significant cell death (P < 0.01) similar to that observed with OGD exposed WT cultures (Figure 4B). In addition, OGD exposure of the WT-OGD CM treated neurons further enhanced the cytotoxicity (p < 0.01) (Figure 4B).

**Figure 5:**
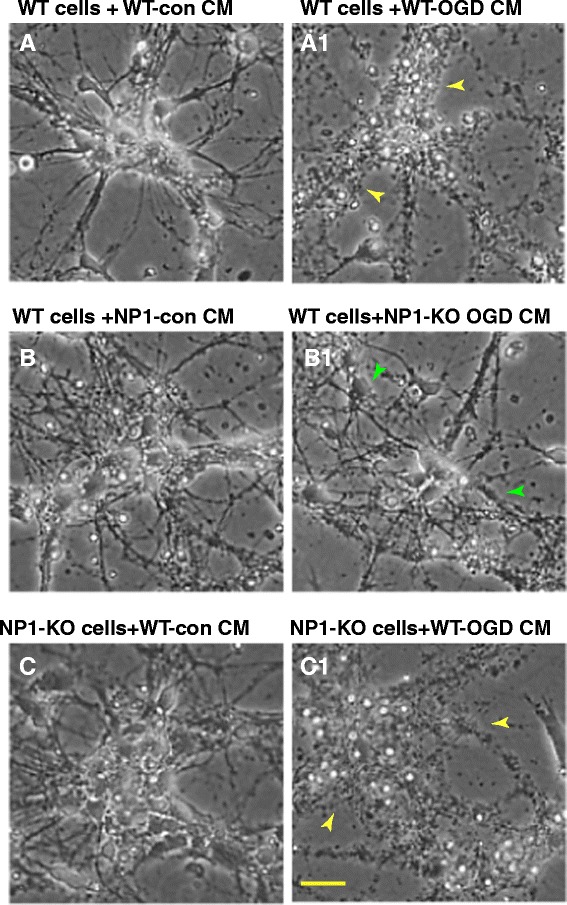
**Absence of NP1 protein in NP1-KO OGD conditioned medium does not cause neuronal death in WT normoxia cortical cultures.** Morphological evidence of degenerated cell bodies and processes reveal that WT OGD CM induced neuronal degeneration in control WT cultures **(A-** **A1)** whereas, OGD conditioned NP1-KO CM did not cause cell death in WT cultures **(B-** **B1)**. In contrast, NP1-KO cortical cultures, which are protected against OGD, showed neuronal death when treated with WT-OGD CM **(C-** **C1)**. Representative light microscopic images are shown, n = 6, Scale, 100 μm.

To specifically examine the role of NP1 in neuronal death, we used CM collected separately from control, OGD-exposed WT (WT-OGD CM) and NP1-KO cortical cultures (NP1-KO OGD CM), concentrated as above, added to separate subsets of WT and NP1-KO cultures at DIV 12, and incubated for additional for 24 h (Figure 5). Most interestingly, we found that WT-OGD CM caused neuronal death (degeneration of cell bodies and processes as shown by yellow arrows) when added to normal WT cultures (Figure 5 A1). In contrast, the CM from OGD exposed NP1-KO (NP1-KO OGD CM) did not cause any degeneration of processes and cell bodies and maintained normal neuronal morphology (shown by green arrows) when added to the normoxia WT cultures (Figure 5B1) similar to control cultures. On the other hand, WT-OGD CM caused neuronal death when added to NP1-KO cultures (Figure 5C1), which otherwise showed neuroprotection against OGD. Our findings confirm the specific involvement of NP1 in neuronal death and neuronal survival in the absence of NP1 protein expression. Taken together, our results clearly demonstrate the role of extracellular NP1 in ischemic neuronal death.

### Presence of NP1 protein in the CM, but not the NP2, is involved in OGD-induced neurotoxicity

**Figure 6:**
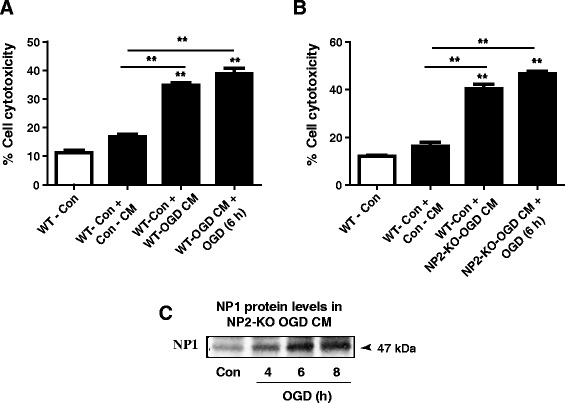
**NP2-KO OGD conditioned medium caused neurotoxicity and death in WT normoxia cortical cultures.** Quantification of cell death by LDH release assay revealed significantly higher percentage of cell death when WT-OGD CM was added to WT control cultures **(A)**. **B)** The NP2-KO OGD CM caused similar extent of cytotoxicity when added to the WT control cultures as compared to control CM (Con-CM). Similarly, the NP2-KO OGD CM in combination with OGD further enhanced cell death. Data shown are mean ± SEM (n = 8 in each group) and repeated two times, **p < 0.01 *vs.* normoxia controls. **C)** Western blot analysis of OGD exposed NP2-KO CM showed OGD time-dependent increased levels of NP1 protein present in the NP2-KO OGD CM, suggesting NP1, but not the NP1, contributes to neuronal death. Representative bands are shown.

To further validate the specificity of NP1 in neurotoxicity, we added CM from OGD-exposed NP2-KO cultures (NP2-KO OGD CM) to a subset of WT normal cortical cultures. As we have observed in Figure 4B and 5A, the LDH release cytotoxicity assay showed significantly increased neurotoxicity (p < 0.01) when the NP2-KO OGD CM was added to normal WT cortical cultures (Figure 6 B), similar to that observed in case of WT-OGD CM (Figure 6A). Western blot analysis of NP2-KO OGD CM revealed increased levels of NP1 protein present in the CM from OGD-exposed NP2-KO cortical cultures. Our results clearly delineate the involvement of NP1, but not the NP2, in ischemic neuronal death following OGD, suggesting the specificity of NP1 in the neuronal injury mechanisms.

### Surface clustering of extracellular NP1 with AMPA GluR1 receptors

**Figure 7:**
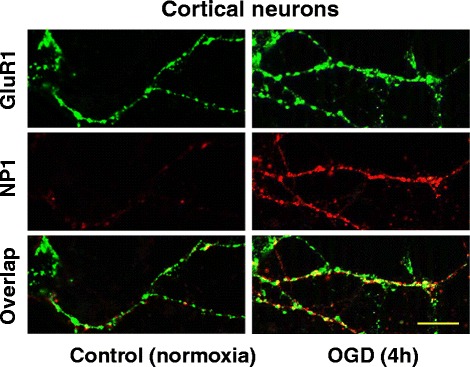
**Extracellular release of NP1 protein following OGD enhances interaction between NP1 and GluR1 at the synaptic sites.** Live immunostaining of DIV 12 primary cortical neurons with NP1 and GluR1 antibodies following OGD (4 h). Immunofluorescence microscopy and merged images show increased number of NP1 (red) and GluR1 (green) co-localized clusters (yellow) at cortical dendrites and axons. Representative images are shown, n = 6, Scale, 100 μm.

Previously we reported that OGD exposure promotes redistribution of AMPA GluR1 receptors at the postsynaptic membrane and significantly increased the NP1-GluR1 interactions at synaptic sites as evidenced by the higher percentage of NP1/PSD-95 co-localization and co-clustering with GluR1 under similar OGD conditions [22]. We asked if extracellular release of NP1 following OGD interacts with GluR1. Live immunostaining with both NP1 and GluR1 antibodies showed intense increase of NP1-GluR1 co-clustering in the dendrites and axons of the OGD-exposed cortical neurons compared to the normoxia controls (Figure 7). Our results suggest a relationship between extracellular release of NP1 following NP1 induction and synaptic clustering of GluR1, which in turn promote OGD-induced neuronal death.

## Discussion

We found increased levels of NP1 protein in the CM from OGD-exposed WT primary cortical neuronal cultures, and this CM caused substantial neurotoxicity when added to control cortical cultures. Most strikingly, the CM from OGD-exposed NP1-KO cortical cultures did not induce neurotoxicity to normal cortical cultures under identical conditions, and that NP1-KO cells maintained characteristic features of healthy neuronal morphology with intact processes. First, we found induction of NP1 following OGD exposure of cortical neurons and neuronal death, whereas, inhibition of NP1 expression by NP1-specific siRNA exerts neuroprotection against OGD. Our findings suggest that NP1, being a secretory protein, after induction following OGD undergoes release from neurons and this extracellular NP1 is involved in ischemic neuronal death. Here, we present evidence that extracellular secretion of NP1 protein following exposure to OGD is necessary to induce ischemic neuronal death, suggesting a new extracellular pro-death function of NP1.

Previously we have reported a role for NP1 in neonatal hypoxic-ischemic brain injury and subcellular localization of NP1 in to mitochondria enhanced mitochondria-mediated neuronal death [6–8]. We also reported interactions of NP1with excitatory AMPA GluR1 receptors and direct regulation of surface GluR1 expression and synaptic clustering of NP1 with GluR1 [22]. These findings suggest the possibility that presynaptic NPR which binds to NP1; allowing NP1 to trans-synaptically attach to the extracellular domain of GluR1 at the postsynaptic specialization, thereby facilitating glutamate binding and, thus, enhancing excitotoxicity. In contrast, NP1-KO neurons showed reduced cytotoxicity by limiting synaptic GluR1 cluster formation due to absence of extracellular NP1 at synaptic sites. We also found that this clustering activity involves physical association between NP1 and GluR1, and that NP1 exhibited profound synaptic co-clustering with GluR1 following OGD [22]. Thus, it is possible that extracellular NP1 may disrupt inter-neuronal synaptic activity, which possibly contributes to the neuronal death in hypoxic-ischemic brain injury *via* clustering with GluR1.

Based on cDNA sequence, NP1 is predicted to be secreted protein [9], raising the possibility that it is present on the neuronal surface that enhances the toxicity of neuronal cells under hypoxic-ischemic stress. We performed a series of experiments to evaluate the importance of extracellular NP1 in hypoxic-ischemic neuronal death. Hypoxic-ischemic neuronal injury is triggered by the activation of glutamatergic excitotoxicity cascade [32] and several downstream cytotoxic pathways [1,33]. It appear from our present findings that NP1 is induced and released following OGD and recruited to surface GluR1 subunits to form clusters at excitatory synapses, and increased NP1-GluR1 interactions sensitize neurons to OGD- induced neuronal death. These members of the long-pentraxin family, NP1 and NP2, are exclusively expressed in the central neurons and are secretory from cells upon pathological stress. To further delineate the role of secretory NPs in neuronal death, we found that it is NP1 protein, not the NP2, is involved in neuronal death as evident by our findings that the CM from the OGD-exposed NP2-KO cortical cultures caused neuronal death when added to the normal cortical cultures similar to that induced by WT-OGD CM. The role of NP1 in neuronal death is also evidenced by the presence of increased amount of NP1 protein in the NP2-KO CM that caused neurotoxicity.

## Conclusions

Collectively, our results argue that extracellular secretion of NP1 following OGD provides a mechanism that potentiates ischemic neuronal death possibly *via* AMPA GluR1-mediated function at the excitatory synapses, and that NP1, not its family member NP2, is involved in neuronal death mechanisms. Our results suggest NP1 as a practical target for preventing ischemic neuronal death.

## Abbreviations

AMPA: α-amino-3-hydroxy-5-methyl-4-isoxazole-propionic acid
CM: Conditioned medium
EBSS: Earl’s balanced salt solution
HI: Hypoxia-ischemia
OGD: Oxygen glucose deprivation
RT-qPCR: Real-time quantitative polymerase chain reaction
NP1: Neuronal pentraxin 1
NP2: Neuronal pentraxin 2
NMDA: N-methyl-D-aspartate
